# Left ventricular remodelling in chronic primary mitral regurgitation: implications for medical therapy

**DOI:** 10.5830/CVJA-2017-009

**Published:** 2018

**Authors:** McCutcheon Keir, Manga Pravin

**Affiliations:** Division of Cardiology, Department of Internal Medicine, Charlotte Maxeke Johannesburg Academic Hospital and University of the Witwatersrand, Johannesburg, South Africa; Division of Cardiology, Department of Internal Medicine, Charlotte Maxeke Johannesburg Academic Hospital and University of the Witwatersrand, Johannesburg, South Africa

**Keywords:** mitral regurgitation, left ventricular remodelling, medical therapy, beta-blocker

## Abstract

Surgical repair or replacement of the mitral valve is currently the only recommended therapy for severe primary mitral regurgitation. The chronic elevation of wall stress caused by the resulting volume overload leads to structural remodelling of the muscular, vascular and extracellular matrix components of the myocardium. These changes are initially compensatory but in the long term have detrimental effects, which ultimately result in heart failure. Understanding the changes that occur in the myocardium due to volume overload at the molecular and cellular level may lead to medical interventions, which potentially could delay or prevent the adverse left ventricular remodelling associated with primary mitral regurgitation. The pathophysiological changes involved in left ventricular remodelling in response to chronic primary mitral regurgitation and the evidence for potential medical therapy, in particular beta–adrenergic blockers, are the focus of this review.

Mitral regurgitation (MR) is caused by failure of adequate coaptation of the anterior and posterior mitral leaflets duringleft ventricular contraction, resulting in various degrees ofregurgitation of blood from the left ventricle (LV) into the leftatrium (LA). The result of this regurgitation is twofold. Firstly,there is a reduction in forward stroke volume (FSV) into theaorta, with subsequent reduction in perfusion. Secondly, there isan increase in LA blood volume during ventricular systole, whichresults in an increase in left ventricular preload, the so-called‘volume overloaded’ state.

MR is classified as either primary (organic) or secondary (functional), and acute or chronic.^1^ Causes of acute MR include infective endocarditis and spontaneous cordal rupture and will not be discussed further in this review. Chronic secondary MR can be ischaemic and/or non-ischaemic in nature and therapies for secondary MR range from medical to surgical.^2^ By contrast, chronic primary MR is predominantly caused by degenerative disease in developed countries,^3^ and rheumatic heart disease (RHD) in developing countries.^4^ RHD is one of the major contributors to the aetiology of heart failure in Africa, where it remains the most common form of acquired cardiovascular disease in children and adults.^4^

Current therapy for patients with severe chronic primary MR, as recommended by the European Society of Cardiology guidelines,^1^ comprises surgical repair or replacement in patients who are surgical candidates, or conservative (i.e. palliative) therapy in patients with very poor left ventricular function who are deemed to be poor surgical candidates. At present, there is no recommendation for drug therapy in patients with any degree of chronic primary MR. However, once heart failure develops, angiotensin converting enzyme inhibitors (ACE inhibitors), beta-blockers and spironolactone may be considered.^5^

Although there have been several recent reviews focusing on ventricular remodelling in ischaemic heart disease, hypertensive heart disease and aortic stenosis, there have been few recent reviews on pathological left ventricular remodelling in patients with primary MR.^6^-^8^ In this review we focus in particular on the pathophysiological changes seen in the myocardium of the LV due to volume overload caused by chronic primary MR. We also discuss medical interventions that may attenuate or reverse the adverse changes seen in chronic primary MR, focusing on data related to the use of beta-blockers in these patients.

## Pathophysiological changes in the LV in chronic primary MR

Primary MR may present acutely, as a slowly progressive disease, or as chronic progressive MR with sudden deterioration related to acute changes in mitral valve anatomy such as a ruptured cord. Acute MR is usually a medical emergency requiring emergent surgery and is not the focus of this review.

Patients with chronic primary MR are often asymptomatic for long periods of time before presenting at a late stage in heart failure. During this period, there is development of progressive left ventricular dysfunction as the LV is remodelled in an attempt to produce an adequate forward stroke volume.^9^,^10^ Five- to 10-year cardiovascular mortality rates vary between 10 and 15%, with a worse prognosis for patients with severe MR.^11^,^12^

**Fig. 1:**
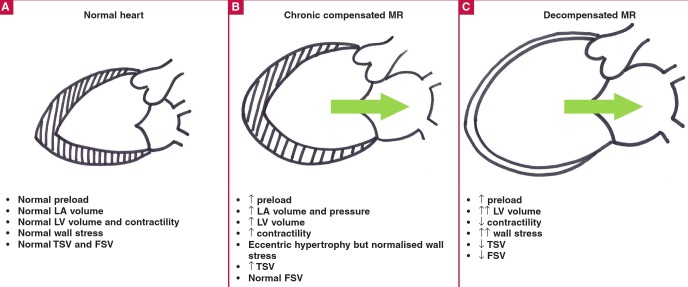
Left ventricular remodelling in chronic primary mitral regurgitation. A: Normal LV is represented on the left. Wall stress is normal. B: Chronic compensation with eccentric hypertrophy and dilatation. The increase in LV volume is compensated for by the increase in wall thickness. Wall stress appears to be normalised by the eccentric hypertrophy. FSV is normal because of increased LV filling. C: Adversely remodelled LV of decompensated chronic MR. The myocardial wall is thin resulting in an increase in wall stress. The arrow indicates severe MR, which becomes more severe with a dilating LV. LA = left atrium; LV = left ventricle; TSV = total stroke volume; FSV = forward stroke volume; MR = mitral regurgitation.

Alterations in the global structure of the LV in response to primary MR have been reviewed in detail previously.^9^ Briefly, MR results in increases in LA volume, a reduction in FSV and an increase in left ventricular preload. By mechanisms that are unclear but are discussed in more detail below, the LV responds to the increased preload by eccentric hypertrophy, with a serial increase in myocyte sarcomeres and myofibril slippage (Fig. 1).^13^-^18^

Eccentric hypertrophy normalises afterload, as estimated by mean systolic stress, compared to patients with aortic regurgitation, leading to a period of so-called ‘compensation’.^19^ However, the hypertrophy that develops is actually insufficient to fully compensate for the wall stress that develops. This is due to inadequate protein synthesis triggered by MR compared to pressure overload,^16^,^20^ and progressive deterioration in myocardial function.^21^

There is no clear explanation for this phenomenon but it has been proposed that the lower systolic load in the case of MR may result in a reduced hypertrophic response at a time when there is a marked demand for an increased stroke volume.^21^ Altered cytoskeletal changes, such as microtubular density, may also play a role.^22^ With time, in the face of inadequate hypertrophy and a dilating LV, systolic wall stress increases [based on the Laplace effect where wall stress (σ) is directly related to the pressure within the ventricle and its radius (Pr), and inversely related to the wall thickness (2h); σ = Pr/2h] due to the increases in LV dimensions and inadequate hypertrophy.^21^,^23^,^24^

Chronic increases in wall stress are detrimental to the myocardium, resulting in activation of a number of complex inflammatory and apoptotic pathways, in a similar manner to heart failure from other causes. Ultimately, there is myocyte loss and sliding displacement of cardiomyocytes, or cell slippage, caused by disruption of the myocardial extracellular matrix (ECM)–integrin linkages.^7^,^25^

Various lines of evidence point to time-dependent changes in the up- and downregulation of remodelling pathways in chronic primary MR.^26^ This process is initiated by diastolic mechanical stretch due to an increase in end-diastolic wall stress, leading to an early increase in reactive oxygen species (ROS) generation, inflammatory cytokine expression and neurohormonal activation, with increases in angiotensin II and catecholamine levels. Early in the remodelling process there is interstitial collagen loss and cell slippage but with time there is myocyte apoptosis and pathological ECM fibrosis.^27^ Chronic decompensated MR ensues, and the LV resembles end-stage dilated cardiomyopathy.

## MR causes mechanical stretch, which triggers mechanoreceptors and activates signal-transduction pathways

Myocardial mechanoreception is currently poorly understood.^28^-^30^ There is no evidence that specialised mechanosensory cells exist in the myocardium and the role of stretch-activated channels in sensing stretch is debatable.^29^ Two systems appear to be particularly important in mechanoreception in the cardiomyocyte: the collagen–integrin–cytoskeleton connections 25,31 and sarcomererelated signalling.^30^

The contraction–relaxation cycle of the myocyte depends on coordinated interaction between the thin actin filament and the thick myosin filament within the myocyte sarcomere.^32^ Actin is bound directly to the Z-disc while myosin is bound indirectly to the Z-disc via the giant elastic protein, titin.^33^,^34^ In the normal heart, titin is responsible for restoring the stretched sarcomere to its resting length following active contraction.^33^,^34^ However, another important role for titin is in mechanoreception and the activation of a number of signal-transduction pathways when there is chronic myocyte stretch.^28^,^29^,^33^,^35^-^37^ It does this by changes in the expression of various genes involved in adaptation to the increased load and, ultimately, to the activation of various maladaptive pathways.^38^-^40^

**Fig. 2:**
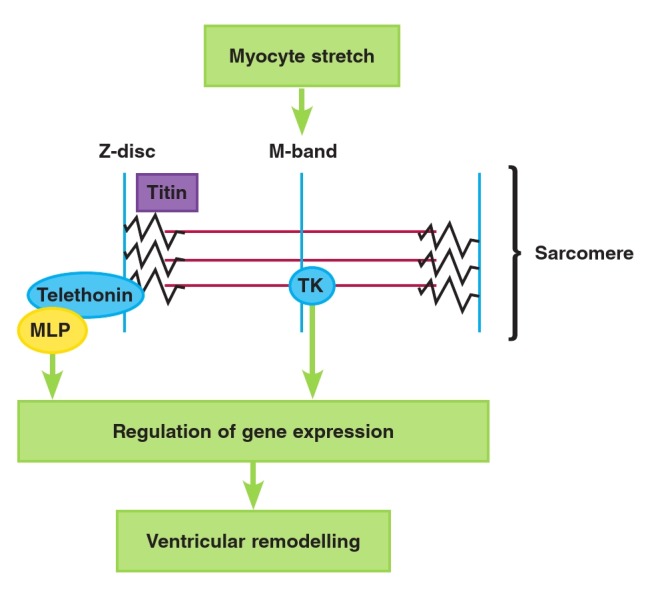
Schematic representation of gene regulation in response to myocyte sarcomere stretch via signal transduction through MLP and TK. MLP = muscle LIM protein; TK = titin kinase. See text for details.

Titin complexes with a number of potential ‘signalosomes’ (a mechanosensative signalling complex), including the Z-disclocalised protein MLP (muscle LIM protein), which has been shown to be responsible for hypertrophy and cardiomyopathy in MLP-deficient animals.^41^,^42^ MLP, aside from its structural role in the Z-disk and its interaction with signal transduction proteins, is able to translocate to the nucleus and thereby act as a transcription factor modifying gene expression, depending on mechanical stretch.^43^ MLP may be responsible for control of other transcription factors coordinating alterations in the expression of genes responsible for ventricular remodelling. Another important titin signalosome that controls muscle gene expression is the sarcomere M-band-associated protein titin kinase (TK), which is activated by myocyte stretch.^38^,^40^ TK may primarily respond to diastolic stretch,^29^ which is particularly relevant in the case of pathological volume overload caused by chronic MR (Fig. 2).

Pathological volume overload-induced mechanical stretch has a number of other effects on the cardiomyocyte. For example, in in vitro^44^ and in vivo^45^ rat experiments, TNF-α is produced by cardiomyocytes, resulting in an inflammatory response to stretch, suggesting that TNF-α is an important component in the pathophysiological response of the myocardium to volume overload. Mechanical stretch also results in the local production of angiotensin II46 and ROS,^47^ which, via transcription factors, such as TRAIL (TNF-related apoptosis-inducing ligand) and NFκB,^47^ result in local increases in pro-inflammatory cytokines, further contributing to activation of remodelling signaltransduction pathways.^48^,^49^ Finally, as discussed in more detail below, mechanical stretch is transmitted through the ECM to cardiomyocyte integrins, which trigger a number of intracellular signal-transduction pathways involved in hypertrophy and apoptosis.^31^,^39^

## Chronic primary MR increases cardiac reactive oxidative stress

ROS play an important role in signal transduction and physiological regulation in vascular and myocardial cells. However, under pathological conditions, such as excessive myocyte stretch or excessive inflammatory signals, ROS have been shown to activate maladaptive remodelling signal-transduction pathways.^50^,^51^ These signal-transduction pathways include (but are not limited to) protein phosphorylation pathways leading to cell growth or apoptosis (depending on ROS levels and other factors); matrix metalloproteinase activation;^52^ cell cycle protein pathways leading to apoptosis; and pathways leading to the activation of inflammatory transcription factors such as NFκB.^53^

**Fig. 3:**
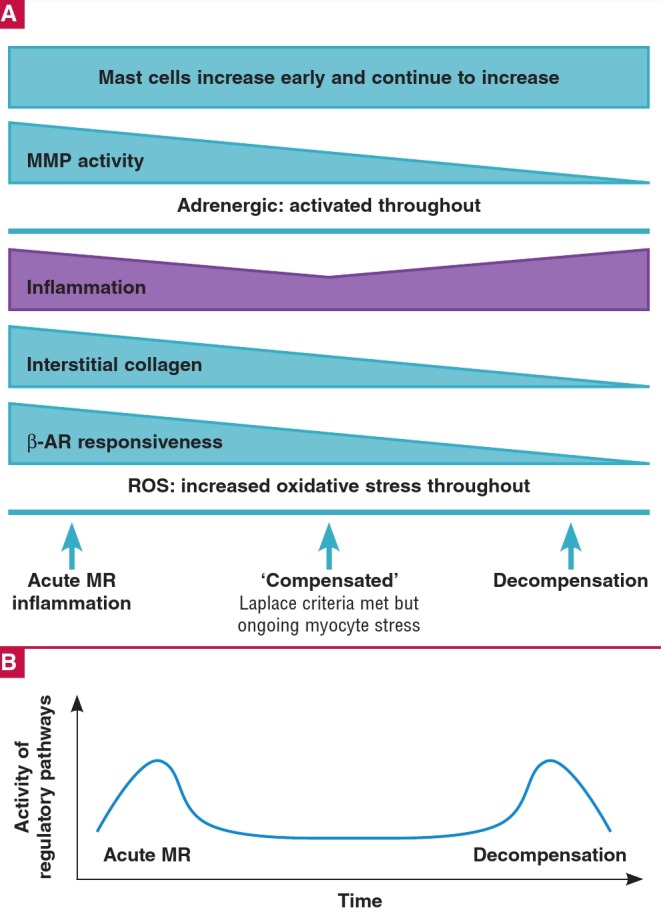
A. Proposed time–dependent changes in various remodelling pathways including changes in measured prevalence of mast cells. B. Proposed overall timedependent changes in remodelling pathway activation. MMP = matrix metalloproteinases; β–AR = β–adrenergic; ROS = reactive oxidative species. See text for details.

ROS are increased in patients with congestive heart failure,^54^ and there is evidence of pathological increases in ROS in patients with chronic isolated MR who still have left ventricular ejection fractions (LVEF) above 60%.^55^ These data suggest that there is an increase in oxidative stress even before the LV starts to develop systolic dysfunction, which supports the notion that wall stress is present throughout the evolution of left ventricular remodelling in primary MR. This oxidative stress appears to be present as long as the volume overload persists (Fig. 3).^56^

## Chronic primary MR triggers an inflammatory Response

Tumour necrosis factor (TNF), interleukin-1 (IL-1) and interleukin-6 (IL-6) are produced by all nucleated cells, including cardiac myocytes.^57^ Cytokines are responsible for beneficial adaptation to short-term stresses, such as haemodynamic overload, within the myocardium. These molecules may play an important role in protecting the heart from oxidative injury and there are several lines of evidence supporting their beneficial role in short-term stress.^58^ However, the role of cytokines in remodelling is complicated and not easily predictable. For example, TNF can have both a protective and an adverse effect on the myocardium, depending on which TNF receptors are activated.^59^ Furthermore, prolonged elevation of tissue cytokines has been found to have deleterious effects on the LV.^60^,^61^

Chronic elevation of cytokines has an effect on left ventricular remodelling by blunting of β-adrenergic signalling^57^ and activation of apoptotic pathways.^62^-^64^ TNF-α also increases cardiomyocyte apoptosis^63^,^65^by activating p38 MAP kinase and NFκB and by down-regulating ERK 1/2 MAP kinase.^66^ Overexpression of TNF has also been shown to increase tissue matrix metalloproteinase (MMP) activity, with the resultant acute loss in myocardial fibrillar collagen and left ventricular dilatation.^67^-^69^ However, with continuing TNF overexpression, there is an increase in tissue inhibitors of metalloproteinase (TIMP-1) expression and reduction in MMP expression, leading to abnormal increases in fibrillar collagen,^67^,^68^ suggesting a time-dependent effect of chronic exposure to elevated myocardial TNF.

Cytokines are elevated in patients with heart failure,^70^ in patients with pressure and volume overload,^71^ and in other forms of heart disease.^72^,^73^Several lines of evidence suggest that the myocardial response to TNFα is similar regardless of aetiology. Gene expression analysis by micro-array suggests that there is a time-dependent inflammatory response to volume overload (Fig. 3).^26^,^45^ Very early after the initiation of volume overload in aortocaval fistula rats, there is a marked increase in the expression of inflammatory pathway genes, followed by relative normalisation during the chronic ‘compensated’ period of volume overload.^26^ This is supported by earlier studies that demonstrate that myocyte stretch induces TNFα secretion from myocytes,^44^,^74^ and mast cell-deficient rats with volume overload were protected from TNFα-dependent left ventricular remodelling.^69^ Furthermore, in humans with compensated chronic primary MR and normal LVEF, there is a down-regulation of inflammatory pathways.^56^

As the LV becomes dilated and dysfunctional, there is an increase in inflammatory pathway gene expression,26 which is supported by clinical work in patients with severe chronic primary MR^71^,^75^ and severe rheumatic aortic regurgitation.^76^Overall, there appears to be a biphasic elevation in the inflammatory response to mitral regurgitation, with early volume overload activating the expression of numerous inflammatory pathways, and decompensation triggering a second inflammatory response (Fig. 3).

## Myocyte loss in chronic primary MR

Apoptosis is activated by several extracellular death signals, including myocyte stretch,^77^ catecholamines^25^,^78^-^80^ and inflammatory cytokines,^57^ and various intracellular death signals.^81^-^85^ These death signals^25^ activate transcription factors,^86^ ultimately resulting in activation of the caspase cascade.^87^ Loss of myocytes will increase the stress on remaining myocytes. This leads to further increases in ROS,^88^ cytokine release,^44^ increases in adrenergic activation,^89^ perpetuating loss of myocytes in a downward-spiraling process. Time-dependent apoptosis of non-myocyte cells has been described in volume-loaded rats^26^ and there is evidence that chronic primary MR causes a reduction in contractile elements.^21^,^82^,^90^ Based on this evidence and evidence from studies in myocardial remodelling due to other causes, it is probable that cell loss is an important component in left ventricular dilatation and dysfunction in chronic primary MR.

## ECM changes in chronic primary MR

Myocyte arrangement and myocardial integrity is highly organised to enable the continuously moving myocardium to produce coordinated contraction, resulting in stroke volume.^91^ The structural integrity is provided by the ECM, which comprises a basement membrane, proteoglycans and glycosaminoglycans, and ECM proteins such as type I, III and V collagen, of which approximately 85% is type I collagen.^7^ This collagen framework serves to maintain cardiac myocyte alignment, without which the myocytes would ‘slip’, altering the shape and size of the cardiac chambers.^91^

The ECM is a highly dynamic part of the myocardium that changes depending on the degree and type of mechanical stress, neurohormonal activation, inflammation and oxidative stress. These stressors on the ECM result in changes in the expression and activation of the proteins responsible for ECM turnover and, ultimately, alterations in collagen deposition and degradation.

MMPs are a heterogeous family of enzymes responsible for the proteolysis of various protein-based extracellular substances. They include the collagenases (MMP-1, MMP-8 and MMP-13), stromelysins (MMP-3 and MMP-10) and the gelatinases (MMP- 2 and MMP-9). They are expressed and secreted into the extracellular space by a variety of cells, including cardiac myocytes, cardiac fibroblasts and macrophages.^92^ However, the roles of each MMP and the control of their activity are not yet clearly elucidated and this is an area of on-going research.^7^ Some studies have demonstrated a correlation between MMP expression and cardiomyopathy phenotypes,^93^-^95^and others have demonstrated that serum levels of MMPs have prognostic value in heart failure.^96^,^97^

TIMPs are low-molecular-weight proteins that bind to the catalytic domain of active MMPs, preventing substrate binding. There are four species of TIMPs with overlapping functions within the myocardium, which are not restricted to MMP inhibition. Other pleomorphic effects have been described. For example, TIMP-2 increases collagen production by fibroblasts, whereas TIMP-3 is responsible for fibroblast apoptosis.^98^

Biological and/or mechanical stimuli trigger various signal transduction pathways, resulting in the production of MMP transcription factors and the secretion of these enzymes into the ECM.^92^,^99^-^101^ Mechanical stimuli, such as stretch,^102^ are transduced through the ECM, which, via collagen–integrin–cytoskeleton connections, are connected to and activate a number of intramyocyte signal-transduction pathways involved in ECM remodelling.^31^,^46^,^103^-^106^ Local ROS, endothelin-1, angiotensin II and catecholamines, via α- and β-receptors, are also responsible for increases in MMP expression.^52^,^89^,^107^,^108^

Cytokines, such as TNFα and IL-1, have been found to increase MMP expression,^67^,^102^ promoting matrix degradation and ventricular dilatation.^7^ On the other hand, MMP-9 production can be suppressed by TGF-β-activated NFκB binding in some experiments,^109^ whereas its expression was up-regulated by angiotensin II-activated NFκB in other experiments.^110^ Angiotensin II^107^ and aldosterone both increase ECM remodelling, mainly through TGF-β,^111^ although the effects of this protein are multiple and often opposing, depending on circumstances.^112^

TGF-β stimulation induces maturation of fibroblasts to myofibroblasts and enhances ECM protein synthesis via induction of TIMP expression and inhibition of certain MMP expression.^111^ However, this is dependent on the load on the myocardium and there is clear evidence that volume overload results in reduction in TGF-β level and loss of interstitial collagen,^113^ whereas pressure overload increases TGF-β.^114^ The result is increased detection of markers of collagen types I and III turnover in the serum,^115^ pathological decreases in interstitial collagen^15^,^116^,^117^ and left ventricular dilatation.

In response to different haemodynamic overloads (pressure versus volume), the ECM undergoes different patterns of remodelling.^27^ Volume overload produces a distinctive loss of collagen fibrils surrounding individual myocytes,^15^,^116^,^118^ with the resultant wall thinning and ventricular dilatation changing the geometrical shape of the LV, whereas excess matrix deposition is observed in pressure overload.^119^,^120^ Despite similar fibrotic molecular pathways and cellular effectors, the pathophysiological mechanisms leading to fibrotic remodelling are different, depending on the load on the heart.^7^ For example, ACE inhibitors reduce remodelling and collagen accumulation in pressure overload,^121^ but not in chronic MR.^15^,^122^ Furthermore, the expression of integrins, which are important in ECM–myocyte connectivity and ECM remodelling, are reduced in MR^113^ but increased in pressure overload.^123^ Similarly, profibrotic TGF-β expression was increased in mice with pressure overload114 but was decreased in dogs with experimental MR,^113^ and expression of PAI-1 was increased in a swine model of early pressure overload^124^ but decreased in chronic MR.^113^

There appears to be a time-dependent increase and decrease in MMP activity during the evolution of left ventricular remodelling in response to primary MR (Fig. 3).^26^,^117^ Myocardial mast cells have been found to be instrumental in increases in MMP activity in early volume overload,^69^,^117^,^125^,^126^ and are increased in number in response to volume overload-induced increases in myocardial TNFα.^45^ In animal models there is an early rise in myocardial MMP levels after the volume-loaded state is created but this seems to normalise after the acute phase.^127^,^128^

MMP gene expression in dogs with isolated MR has confirmed that, at four months, there was down-regulation of a number of non-collagen genes important in ECM structure, down-regulation of pro-fibrotic connective tissue growth factor and plasminogen activator, and down-regulation of numerous genes in the TGF-β pathway.^113^ However, MMP-1 and MMP-9 gene expression was still markedly increased in these dogs with compensated MR compared with controls.^113^ As the LV started to dilate in dogs with chronic myxomatous mitral valve disease, MMP-9 levels decreased.^129^

Over time, there are characteristic changes in the MMP/TIMP ratio, enabling the ventricle to initially increase compliance in the acute and compensated phases of MR. However, at some point (the ‘transition’ point) there is excessive degradation of the ECM, leading to the decompensated and dilated LV.^27^,^130^ What controls the steady deterioration in the myocardium in response to volume overload is not clear and appears to be complex. In the early stages of volume overload, there are decreases in ECM deposition (which contrasts with the picture in pressure overload),^113^ but late in the progression of the dilating volumeloaded heart, an increase in perivascular collagen deposition has been noted,^26^,^126^ which may reduce ventricular compliance and promote systolic dysfunction.^27^

## Chronic primary MR activates the neurohormonal system: implications for beta-blocker therapy

Patients with chronic primary MR demonstrate LV systolic dysfunction even before a reduction in LVEF occurs.^131^,^132^ As with heart failure due to any other cause, chronic MR results in activation of the neurohormonal system and inflammatory cascade at both systemic and local levels.^133^-^135^ With neurohormonal activation, myocardial angiotensin II plays an important role in the regulation of cell proliferation, apoptosis, inflammation and production of mediators of remodelling such as platelet-derived growth factor and MMPs.^136^ Persistent angiotensin receptor-1 activation by angiotensin II not only results in the generation of ROS but also alterations in protein synthesis via tyrosine kinase receptor activation and MAP kinase signalling.^137^ Furthermore, angiotensin II-activated ROS act as second messengers that also have effects on inflammation and cell growth.^138^ Angiotensin II also acts on the sympathetic nerve endings in the myocardium to facilitate catecholamine release.^139^,^140^

Long-term increases in myocardial angiotensin II levels increase local TGF-β, with the resultant increases in activation of genes involved in ECM production via nuclear translocation of NFκB.^110^,^141^ Unlike the pressure-overloaded heart where there is progressive fibrosis,^142^ the increase in myocardial angiotensin II in volume overload results in an increase in ECM turnover with loss of interstitial ECM.^143^ Despite the clear link between angiotensin and remodelling in heart failure, to date there has been little clinical evidence to support the role of medical therapy directed against angiotensin in subjects with chronic organic MR.^15^,^144^-^146^ This may be explained by the fact that ACE inhibitors reduce the breakdown of bradykinin, which has been implicated in the initial increase in MMP activity and collagen breakdown seen in volume overload.^143^

Three types of β-adrenergic receptors (β-ARs) are known to exist in the myocardium: β1, β2 and β3, with an approximate ratio of 80:17:3.^147^ β1 and β2 are important in the regulation of myocyte excitation–contraction coupling.^80^ β1-AR is the predominant receptor subtype expressed in the heart and, like other β-ARs, its stimulation results in G-protein-coupled activation of the adenyl cyclase–cAMP–protein kinase A (PKA) signalling cascade. This leads to activation of a number of subcellular pathways important in cardiomyocyte contractile function, including calcium channel activation and troponin I phosphorylation. By contrast, β2-AR signalling has negative effects on adenyl cyclase activation and the subsequent G-protein-activated ionotropic response.^80^ β3-AR appears to be important in protection from hypertrophic and fibrotic remodelling by preserving NO/cGMP signalling during cardiac stress.^148^

The β-adrenergic receptor system plays an important role in the pathogenesis of myocardial remodelling and heart failure.^84^ The exact mechanisms are unclear but it has been known for decades that chronically increased plasma catecholamines can lead to heart failure.^149^,^150^ In dogs with chronic primary MR, there is activation of the adrenergic system,^89^,^108^ and recent gene array data in chronic primary MR patients with preserved LVEF demonstrate increased expression of genes involved in β-adrenergic signalling.^56^ This indicates that the adrenergic system is activated during the compensatory phase of MR and supports the concept that blocking these pathways may reduce their adverse consequences. However, with transition to decompensation there is a reduction in adrenergic responsiveness.

In patients with systolic heart failure (HF), several studies in the last three decades show that â1-receptor density^151^ and its mRNA^152^,^153^ are reduced while â2-receptor density remains unchanged.^154^ Similarly, in animals with HF due to chronic volume overload, â1-AR responsiveness is reduced due to neurohormonal activation (Fig. 3).^47^,^155^ These changes in â1-AR expression are caused by sustained adrenergic activity, causing an increase in the expression and activity of GRK 2 (G-proteincoupled receptor kinase; formerly called â-ARK or â-agonist receptor kinase), resulting in â1-AR being phosphorylated and labelled for desensitisation, internalisation and recycling.^156^ The result is a reduction in the density of â1-ARs and a reduced propensity for myocyte activation by chronic â1-receptor activation, which may protect the myocyte from long-term catecholamine toxicity.^80^

## Beta-blocker therapy improves β1-AR signalling and clinical outcomes in HF

Chronic β1-AR activation causes a number of detrimental effects, ultimately resulting in changes in the ECM and cell loss from necrosis and apoptosis,^25^ which in turn leads to cardiac dilatation and failure.^157^ However, the intracellular pathways responsible for these final acts are unclear.^25^ What is clear is that β1-AR antagonists improve clinical outcomes in patients with systolic heart failure and improve cardiac function and myocardial remodelling.^158^

Most β-adrenergic blockers are antagonistic to β-ARs (whether β-1, β-2 or β-3) by occupying the receptor and preventing signal transduction via G-protein activation. Importantly, there is an up-regulation of β1-AR expression and improvements in receptor sensitivity, resulting in reversal of cardiac remodelling.^147^ However, the pharmacological and clinical effects of these agents vary quite considerably. Cardioselective beta-blockers (metoprolol, bisoprolol and atenolol, for example) have a greater affinity for β1-ARs than β2-ARs, whereas carvedilol binds β1-ARs more than β2-ARs and has vasodilatory effects, via nitric oxide and α1-receptor blockade.

There are other important differences between carvedilol and other beta-blockers. For example, metoprolol upregulates cardioprotective β3-AR expression, whereas carvedilol does not.^159^,^160^ Carvedilol has antioxidant and antiproliferative properties^161^-^163^ and differs from metoprolol in its effects on haemodynamics, left ventricular function and β1-AR expression.^164^,^165^ Carvedilol^166^ and bisoprolol^167^ have also been shown to improve right ventricular (RV) ejection fraction, attenuate RV dilatation and reduce pulmonary artery hypertension in patients with ischaemic and non-ischaemic dilated cardiomyopathy. Although there are no recent confirmatory studies, these improvements in RV function may be related to reductions in RV afterload and/or improvements in RV contractility.^166^,^167^ By contrast, short-term (two-week) metoprolol did not improve RV function in patients with moderate-to-severe degenerative MR.^168^

Clinical support for β-adrenergic receptor blocker therapy in patients with heart failure is well known,^158^,^169^,^170^ with some data suggesting that patient outcomes are better with carvedilol than the immediate-release form of metoprolol.^171^

Several mechanisms for the improvement in outcomes with β1-receptor blockade have been proposed,^84^ including antiarrhythmic properties;^172^ improved β-adrenergic signalling by cardiac β-AR upregulation;^80^ free-radical scavenging;^161^ improvements in calcium cycling by the sarcoplasmic reticulum;^83^,^173^ bradycardia reducing myocardial work, mechanical stress,^174^ and prolonging diastolic calcium uptake and cycling by the sarcoplasmic reticulum; inhibition of the renin– angiotensin–aldosterone system; and there is growing evidence that β-antagonists, in particular carvedilol,^162^,^163^ directly reduce apoptosis^78^,^175^-^177^ and collagen loss by MMP activation.^178^

## β1-AR blockade in MR counters adverse adrenergic effects

Since MR leads to a reduction in forward stroke volume, it is hypothesised that the adrenergic system is activated to maintain systemic blood pressure and perfusion, and blockade of the adrenergic system should limit adverse left ventricular remodelling. There is evidence that chronic primary MR results in excessive activation of the sympathetic nervous system, with increases in myocardial catecholamine levels,^89^,^118^,^134^ similar to heart failure from other causes.^179^

Tallaj et al.^118^ demonstrated that β-AR blockade with extended release metoprolol succinate attenuated angiotensin II-mediated norepinephrine and epinephrine release in the myocardium of dogs with ‘subacute’ (two to four weeks’ duration) isolated MR. Similarly, Hankes et al.^89^ demonstrated that norepinephrine release into the cardiac interstitium was significantly higher in dogs with subacute MR, which was reduced by β1-AR blockade. In an earlier study by Tsutsui et al.^90^ in ‘chronic’ (three months) canine MR, the β1- AR blocker atenolol improved left ventricular function, which was associated with improvement in contractile function of isolated cardiocytes and an increase in the number of contractile elements. This was supported by a similar study by Nemoto et al.,^145^ which showed that only when a β1-AR blocker was added to an ACE inhibitor did forward stroke volume and cardiac contractility return to normal. Recently, Trappanese et al.^180^ demonstrated an improvement in β3-AR expression and β3-NO-cGMP coupling with chronic therapy with metoprolol in dogs with primary MR. Since β3-AR is cardioprotective, this may partially explain the potential beneficial effects of β1-AR blockade in primary MR.^159^

## Beta-blockade therapy for chronic primary MR

At present there is no proven medical therapy for chronic primary MR. Surgery is the mainstay of treatment for severe MR1 but carries peri-operative risk, and patients are potentially subjected to a life-time risk of anticoagulation if they undergo mitral valve replacement. Many patients in the developing world are from poor and rural backgrounds where access to regular medication and regular anti-coagulation assessment is difficult. A medication that could limit or even reverse left ventricular dysfunction associated with the volume-loaded state of chronic severe MR would be extremely beneficial to these patients, even if only to delay the need for surgical intervention. This would especially be true in women of child-bearing age. Warfarin is teratogenic and many women with prosthetic valves have complicated pregnancies related to the teratogenic effects of warfarin or the risks related to bleeding during pregnancy.

Persistent, and often worsened, postoperative left ventricular dysfunction is a major cause of morbidity and mortality in these patients,^133^ although this is not a universal finding, especially when patients are referred for early surgery.^181^-^183^ Nevertheless, a means to improve left ventricular function in the peri-operative period might improve the postoperative morbidity and mortality rates associated with left ventricular dysfunction.

Current guidelines^1^,^184^ recommend surgery for patients with severe pulmonary hypertension on presentation or a progressively dilating LV, even if they are asymptomatic. However, timing of surgery is uncertain^185^,^186^ and there is no clear guideline as to the urgency of the surgery in asymptomatic patients without overt left ventricular systolic dysfunction (LVEF < 60%). Several studies support early surgery for chronic primary MR.^187^–^189^ Enriquez–Sarano et al.^12^ showed that patients with an effective regurgitant orifice area of at least 40 mm^2^, as assessed by echocardiography, should promptly be considered for cardiac surgery. Barbieri et al.^190^ found that asymptomatic patients with evidence of pulmonary hypertension ( > 50 mmHg at rest) should undergo prompt surgery. However, there is also evidence that asymptomatic patients with severe MR can be followed until they become symptomatic or demonstrate echocardiographic signs of left ventricular dysfunction.^132^,^191^

How these patients should be managed in the interim is unclear but it is important that patients are not left untreated until irreversible left ventricular remodelling has taken place. In resource–limited hospitals, patients who do not need emergency surgery often wait several months before they undergo surgery, by which time there has been progressive advancement of ventricular remodelling, leading to permanent impairment of function. Medical therapy that could reverse or at least attenuate LV remodelling may improve outcomes in these patients.

To date, there is little evidence to support medical therapy in the treatment of patients with organic valve disease,^1^,^119^,^192^ or in the reversal or attenuation of LV remodelling, which may delay the need for surgery in asymptomatic patients.^193^ Vasodilator therapy, which reduces peripheral vascular resistance and left ventricular afterload,^119^ has generally not improved outcomes in patients with MR or in experimental MR canine models.^15^,^122^,^145^,^194^ Although several small studies from the 1970s to the 1990s showed benefit of vasodilator therapy in acute MR,^144^ small human studies from the same period failed to show long–term benefit.^195^ One retrospective study, however, demonstrated an improvement in echocardiographic LVEF in patients treated with after load–reducing agents.^146^ However, there are no large randomised studies assessing long–term vasodilator therapy, including ACE inhibitors, in humans.

There is some clinical evidence to support the concept that β–AR blockade may attenuate remodelling in patients with primary MR (Table 1). Stewart et al.^196^ recruited 25 patients with moderate or severe degenerative MR and randomly assigned the participants to the β1–AR blocker metoprolol, or placebo for approximately two weeks. Left ventricular function was assessed at baseline and on study completion by cardiac magnetic resonance imaging. They found that the β1–AR blocker resulted in a decrease in left ventricular work and an increase in forward stroke volume.^196^ Mitral annular dimensions also appeared to improve over the two–week period in the same cohort of patients.^197^

**Table 1:** Studies of beta-blocker therapy and left ventricular function in primary MR

*Authors*	*Year*	*Subject*	*Cause of MR*	*Number treated with BB*	*Type of study*	*Control*	*Type of BB*	*Duration of BB*	*Outcome measures*	*Favours BB*
Tsutsui et al.^89^	1994	Dog	Experimental chordal rupture	n = 6	Case Controlled	n = 6	Atenolol 50mg daily	3 months	Cardiocyte contractility, myofibrillar density	+
Nemoto et al.^144^	2002	Dog	Experimental chordal rupture	n = 11	Longitudinal	NA	Atenolol 100 mg Daily	3 months	Haemodynamics, LV Function	+
Tallaj et al.^117^	2003	Dog	Experimental chordal rupture	2 weeks MR+BB: n = 6 4 weeks MR+BB: n = 8	Case Controlled	Normal: n = 8 2 weeks MR: n = 8 4 weeks MR: n = 6	Metoprolol Succinate	4 weeks	RAAS activation	+
Hankes et al. 88	2006	Dog	Experimental chordal rupture	4 weeks of MR+BB = 8	Case Controlled	Normal = 6 Untreated MR = 6	Metoprolol succinate 100 mg Daily	4 weeks	NE release into cardiac interstitium	+
Oh et al. 145	2007	Human	71% degenerative	n = 134	Retrospective Cohort	NA	Not ascertained	1–88 Months	Echo LVEF	–
Pat et al. 199	2008	Dog	Experimental chordal rupture	n = 11	Case Controlled	n = 10	Metoprolol succinate 100 mg twice daily	4 months	LV remodelling by MRI and echo; cardiomyocyte function	Improved cardiomyocyte function and BB receptiveness but failure to attenuate remodelling
Sabri et al.^107^	2008	Dog	Experimental chordal rupture	n = 6	Case Controlled	Normal = 6 Untreated MR = 6	Metoprolol succinate 100 mg Daily	4 weeks	LV remodelling by echo; interstitial collagen quantification; FAK signalling (integrin signalling)	BB reduced FAK tyrosine phosphorylation but no change in remodeling parameters; BB reduced epicardial collagen loss but not endocardial collagen loss
Varadarajan et al.^197^	2008	Human	LVEF > 55% + ‘severe MR’	n = 218	Retrospective observational cohort study	n = 614	Not stated	8 years	Mortality	+
Stewart et al. 195	2008	Human	MVP	n = 25	Double-blind cross-over study	NA	Metoprolol to a maximum 190 mg daily	14 days	MRI EF	–
									LVEDV	–
									LVESV	–
									LV ‘work’ (CO)	+
Ahmed et al. 198	2012	Human	MVP	n = 19	RCT	n = 19	Toprol XL		MRI LVEF	+
							25–100 mg		MRI LVESV	–
							Daily		LV longitudinal strain rate	–
Pu et al. 200	2013	Rat	Experimental leaflet disruption	n = 43 ‘Long-term’ BB in 19	RCT	n = 44	Carvedilol (1 200 ppm)	36 weeks	Echo only LV dimensions LVESV and mass index FS and EF Survival probability	–
Trappanese et al.^179^	2015	Dog	Experimental chordal rupture	n = 8 (MR + BB)	Case Controlled	Normal = 10	Metoprolol succinate	4 weeks	Activation of β3AR/ NO-cGMP signalling	+
						Untreated MUntreated MR = 8R = 8	100 mg Daily		β3-AR expression		+

BB = beta-blocker; MVP = mitral valve prolapse; RCT = randomised controlled trial; MR = mitral regurgitation; NE = norepinephrine; + indicates that the study favoured BB therapy in primary MR; – indicates that the study did not favour BB therapy in primary MR; LV = left ventricle; LVEF = left ventricular ejection fraction; LVEDV = left ventricular end-diastolic volume; LVESV = left ventricular end-systolic volume; CO = cardiac output; RAAS = renin–angiotensin–aldosterone system; echo = echocardiogram; MRI = magnetic resonance imaging; ppm = parts per million.

A retrospective observational study involving 895 patients in California showed that participants on β1–AR blocker therapy with severe MR and normal left ventricular function had a significantly lowered mortality hazard, regardless of the presence of hypertension or coronary artery disease.^198^ Ahmed et al.^199^ published the results of the first randomised, controlled phase IIb trial of beta–blockade in patients with chronic degenerative MR. Thirty–eight asymptomatic patients with moderate–tosevere isolated MR were randomised to either placebo or longacting metoprolol for two years. Cardiac magnetic resonance analysis showed that patients randomised to the β–AR blocker had significantly better LVEFs after two years of therapy.

By contrast, there are several pre–clinical and clinical studies demonstrating that beta–blocker therapy actually worsens left ventricular dimensions and function in chronic primary MR (Table 1).^108^,^146^,^197^,^200^,^201^ A recent, longer–term (23 to 35 weeks) study in rats found that echocardiographic measures of left ventricular remodelling were not improved by carvedilol.^201^ In fact, left ventricular dimensions, ejection fraction and survival were significantly lower with long–term carvedilol use.

Similarly, in a four–month dog model, extended–release metoprolol succinate failed to attenuate the adverse global left ventricular remodelling and ECM loss, but did preserve cardiomyocyte function.^200^ Interestingly, all dogs treated with β–1–receptor blocker (n = 6) survived to four months, whereas only five out of nine of the untreated dogs survived to four months. Similarly, Sabri et al.^108^ found that, despite reductions in interstitial collagen degradation and reductions in adverse remodelling–related intracellular signalling, extended–release metoprolol succinate failed to attenuate left ventricular dilatation or decline in left ventricular function.

A retrospective echocardiographic study in 134 human subjects with moderate–to–severe MR (67% degenerative and 20% ‘non–specific thickening’) found that patients exposed to beta–adrenergic blockade developed worsening of their ejection fraction over a mean of 20 months of follow up.^146^ Finally, despite improvements in left ventricular work and annular dimensions,^197^ in patients treated over a short period with metoprolol, there were significant increases in left ventricular end–systolic and end–diastolic volume with no significant change in LVEF or regurgitant volume.^196^

At the present time, there are no recommendations regarding medical therapy in chronic primary MR. Afterload reduction has not consistently been shown to improve long–term outcomes.^15^,^122^,^145^,^146^,^195^ Data from heart failure trials^158^,^169^,^170^ as well as from animal models^90^ and human trials^199^ suggest a role for beta–blockade in MR, however, other studies do not support this.^108^,^146^,^197^,^200^,^201^ The reasons for the discrepancies in these findings are unclear but some explanations can be proposed.

Firstly, the studies have been performed in different experimental models and at different stages in the evolution of MR–related left ventricular remodelling. Many of the experiments performed thus far have been in animal models with controlled formation of volume overload showing that early introduction of beta–blocker therapy^89^,^90^,^118^ may be beneficial, and this is supported to some extent by the work of Ahmed et al.^199^ in humans. However, there appears to be a time–dependent pattern during remodelling of the LV in chronic primary MR.

Early after the development of MR there is a marked increase in inflammatory and neurohormonal response to the acute volume overload.^26^,^113^ A period of compensation and a relatively normal inflammatory response appears to follow until the late decompensated stage is reached, when adverse pathway activation seems to increase.^26^ Depending on when in this evolution of left ventricular remodelling the studies to date have been performed, there may be discrepancies in the findings with regard to the impact of beta–blockade on left ventricular remodelling. Beta–blockade may have more impressive effects if used early in the evolution of volume overload–related left ventricular remodelling but it may be less effective later on.

Secondly, various beta–adrenergic agents have been tested under different circumstances. Compared to the impressive beneficial results in heart failure patients with the mixed adrenergic blocker, carvedilol,^158^,^202^ Pu et al.^201^ demonstrated that this drug caused worsening left ventricular dimensions and function in animal subjects with primary MR. By contrast, treatment of dogs with the selective beta–blocker atenolol improved left ventricular remodelling,^90^ whereas treatment with metoprolol in animals and humans have had mixed results.^108^,^180^,^196^,^197^,^199^,^200^

Thirdly, an important question is whether patients presenting in more advanced stages of left ventricular remodelling will respond to anti–remodelling therapy or whether the wall stresses determined by the Laplace law will outweigh any potential beta–blocker effect. In this regard, the findings of Pat et al. 200 are interesting because although there were improvements in cardiomyocyte contractility and beta–receptor responsiveness, left ventricular remodelling was not attenuated by metoprolol. It was postulated that β–AR blockade failed to preserve interstitial collagen loss and therefore failed to prevent ongoing myocyte slippage. There appears to be a strong early adrenergic response to chronic primary MR,^56^ and beta–blocker therapy before onset of left ventricular dilatation may have more benefit. Lastly, in patients with rheumatic mitral valve disease, there is a possibility that rheumatic fever causing rheumatic carditis may have long–lasting effects on the myocardium, attenuating reverse remodelling by beta–blockers.

## Conclusion

Left ventricular remodelling in response to the volume load created by chronic primary MR is a complex process that stems from excessive diastolic stretch of myocytes. Excessive stretch triggers activation of numerous signal transduction pathways, resulting in an initial adaptive remodelling process in the form of eccentric hypertrophy. However, chronic activation of these pathways results in abnormal increases in ROS, catecholamines, angiotensin II and inflammatory cytokines. This is followed by a transition to adverse remodelling involving cardiomyocyte apoptosis and interstitial collagen loss, common to all forms of heart failure.

Limited data do not support the routine long–term use of afterload–reducing agents for the treatment of chronic primary MR. By contrast, there is pre–clinical data demonstrating that β–AR blockade reverses remodelling caused by the volume overload of chronic primary MR, and there are some recent clinical data to support this hypothesis. However, some studies demonstrated worsening left ventricular remodelling with betablocker therapy. Whether these contrasting outcomes are related to differences in beta–blockers, varying experimental models or differences in timing of therapy will need clarification. Ultimately, further studies are required to elucidate the exact mechanisms involved, and large randomised clinical trials are needed to clarify the role of these agents for patients with chronic primary MR.
